# The COVID‐19 pandemic caused gender‐specific declines in knee surgery rates in Sweden from 2020 to 2021

**DOI:** 10.1002/jeo2.70030

**Published:** 2024-10-03

**Authors:** Michael Axenhus, Martin Magnéli

**Affiliations:** ^1^ Department of Orthopaedic Surgery Danderyd Hospital Stockholm Sweden; ^2^ Department of Clinical Sciences, Danderyd Hospital Karolinska Institutet Stockholm Sweden

**Keywords:** COVID‐19, knee arthroplasty, knee surgeries, surgical trends, Sweden healthcare

## Abstract

**Purpose:**

Changes in knee surgery incidence are important factors for stakeholders and healthcare providers. The aim of this study was to examine trends and patterns in knee surgeries in Sweden from 2010 to 2022. The study focuses on gender‐specific and overall rates of knee surgeries.

**Methods:**

The analysis is based on a data set sourced from national healthcare records. The data was stratified based on surgical rates and categorized by gender, year and the specific knee arthroplasty technique used. We tracked year‐to‐year changes in surgical rates to identify overarching patterns. We used Poisson regression to predict future trends. Comparisons were made between various surgical subcategories, such as those with and without cement in knee arthroplasty surgeries.

**Results:**

In 2010, the rate of knee surgeries per 100,000 person‐years was 518.7 for males and 448.0 for females. These rates exhibited fluctuations over time, reaching their lowest point in 2020, attributed to the pandemic's disruption of elective procedures, with 386.4 surgeries per 100,000 males and 386.3 surgeries per 100,000 females. A resurgence was observed in 2022. The rates of primary knee arthroplasty increased, with a male rate of 106.2 and a female rate of 150.7 surgeries per 100,000 inhabitants in 2010, rising to 126.8 for males and 166.2 for females in 2022.

**Conclusion:**

This comprehensive nationwide open‐source data analysis of knee surgeries in Sweden shows that the COVID‐19 pandemic significantly impacted knee surgery rates in Sweden, causing a notable decline in 2020, followed by a resurgence in 2022. Furthermore, while men had higher surgery rates than women, they experienced a larger decline in the incidence of knee surgeries compared to women. Understanding these trends is crucial for stakeholders and healthcare providers to improve resource allocation, address gender disparities, and maintain the resilience of surgical services in the face of disruptions.

**Level of Evidence:**

Level III.

## Graphical abstract

The COVID‐19 pandemic led to gender‐specific declines in knee surgery rates in Sweden from 2020 to 2021. While men generally had higher surgery rates, they experienced a larger decline compared to women during the pandemic. Future trends indicate changes in surgery trends and incidence during the coming years.

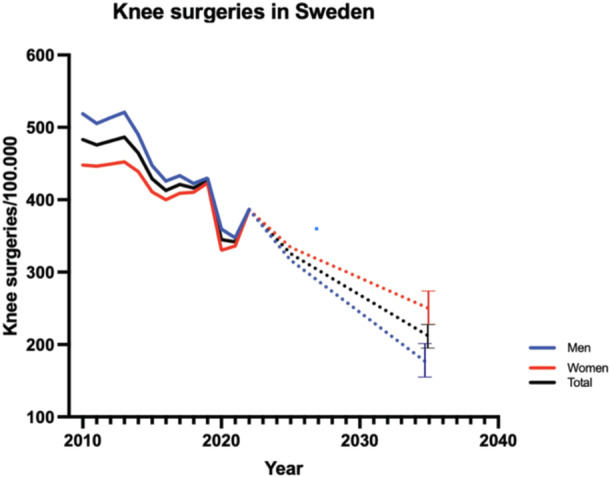

**Abbreviation**
COVID‐19: coronavirus disease 2019

## INTRODUCTION

Knee surgeries, in particular arthroplasty, play a pivotal role in modern healthcare, offering relief and improved quality of life to individuals grappling with degenerative joint conditions. Together with hip osteoarthrosis, knee osteoarthrosis represents a significant global disability ranking slightly below that of diabetes [19]. Knee arthroplasty remains one of the most common orthopaedic surgical interventions and costs for knee arthroplasty are rising [33]. Over the past decade, Sweden, like many nations, has witnessed significant evolution in the landscape of surgical interventions for knee‐related issues. There have been attempts to describe the future need for knee arthroplasty in Sweden, although studies focus on the overall increase in surgery numbers and do not take into account changes in surgical techniques and changing surgical indications [21, 25]. Understanding the dynamics of these surgeries is of paramount importance for healthcare planning, especially in light of the unprecedented disruptions caused by the coronavirus disease 2019 (COVID‐19) pandemic. An ageing population also puts pressure on stakeholders to increase healthcare capacity to meet demand [35].

It is evident that the past decade has witnessed a series of intriguing developments in knee surgery techniques and preferences in Sweden [24, 26]. Surgical rates have exhibited fluctuations, influenced by factors ranging from advancements in medical technology and changes in patient factors to the unforeseen challenges posed by the global pandemic [6, 22, 24]. The evolution of primary knee arthroplasty surgeries, both in terms of sex‐specific variations and surgical subcategories, could influence surgery incidence [17, 32]. Understanding the evolving landscape of knee surgeries, in particular knee arthroplasties, is therefore pivotal to ensuring that individuals in need of these procedures receive timely and effective care. We hypothesize that there has been a significant increase in knee surgery and knee arthroplasty incidence during the last few years. No estimation for the future development of various knee surgeries in Sweden during the coming years exists. Therefore, the aim of the study was to analyse the overall trends of different knee surgery procedures with a focus on surgery technique and population group affected using open‐source database analysis.

## METHODS

### Ethical considerations

This research is based on publicly available aggregated healthcare data, and no personally identifiable information was utilized. As a result, ethical approval and informed consent were not required for this study.

### Data collection

This retrospective population‐based study used population census data from Sweden Statistics (www.scb.se) and information about knee arthroplasty from the National Board of Health and Welfare's National Patient Register (www.socialstyrelsen.se). The data set covers the period from 2010 to 2022 and includes detailed information on all knee surgical procedures, defined per healthcare intervention coding known as Classification by Healthcare Procedure (CHP). We extracted data based on surgical rates, categorized by sex, year and specific surgical types from 1 January 2010 to 31 December 2022. The data were stratified into two groups on age below of above 65 years of age.

### Study population

The study population comprised individuals residing in Sweden who underwent knee arthroplasty surgery (total or uni) between 2010 and 2022. The timeframe chosen allowed for a comprehensive analysis of temporal trends in knee arthroplasty with a long enough time span for future trend projection.

Inclusion criteria:

1. Individuals residing in Sweden at any time from 1 January 2010 to 31 December 2022.
2. Individuals who have undergone primary total‐, unicondylar‐ or hybrid technique knee arthroplasty with or without cement in Sweden.

Exclusion criteria:

1. Knee surgery due to pathological fracture, infection or aseptic loosening.
2. Knee arthroplasty revision.
3. Knee level amputations.
4. Primary meniscectomies, osteotomies and ligament repair surgeries.

The specific included procedural codes are provided as Supporting Information (Table S1).

### Data analysis

The overall annual incidence of knee arthroplasty per 100,000 person‐years was calculated and stratified according to sex and age groups. Data were categorized primarily by sex, age and surgical procedure. The incidence rates were calculated by dividing the number of knee arthroplasties by the corresponding age‐specific population estimates obtained from Statistics Sweden [29]. The mean incidence was calculated from the number of knee arthroplasties and the study population.

The knee arthroplasty incidence was determined by dividing the annual count of new knee arthroplasties by the total population of Swedish residents. We opted for modelling incidence rather than the total number of knee arthroplasties to account for potential shifts in the population composition. Future trend predicting was calculated using Poisson regression modeling which utilized the estimated incidence as the outcome variable, with the calendar year serving as the input factor. For future trend progression analysis, we chose 65 years of age as a cut‐off point due to the higher incidence of arthritis and bone‐related illnesses in this population. Year‐to‐year changes in surgical rates were closely examined to identify fluctuations and evolving patterns. This analysis allowed for a deeper understanding of how surgical rates evolved over time. The data set was parsed to distinguish between male and female surgical rates. This gender‐specific analysis aimed to identify any notable variations and trends specific to each gender. 95% confidence intervals (CIs) are used were applicable. A *p* value of <0.05 was considered significant.

## RESULTS

**Figure 1:**
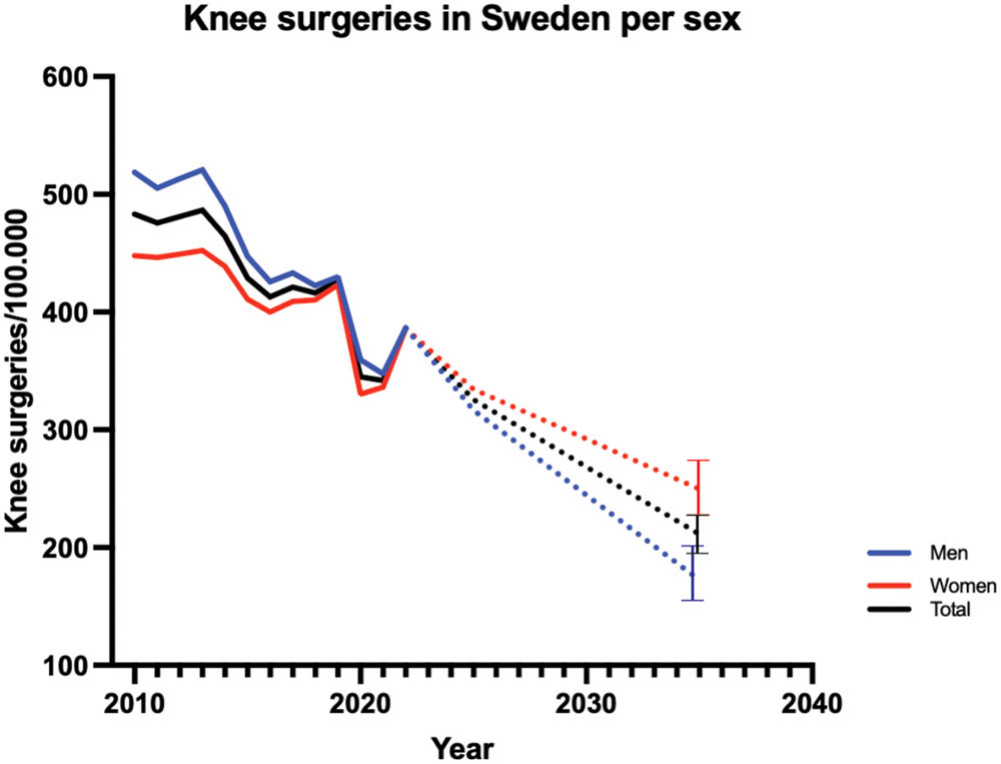
Incidence of knee surgery among men and women in Sweden 2010–2022. Future trend is indicated by dotted lines. Bars indicate 95% CI. CI, confidence interval.

During the study period, there was an overall decline in the incidence of knee surgeries. In 2010, the rate of surgeries per 100,000 person‐years was 518.7 for males and 448.0 for females. The lowest point was noted in 2020, with a significant resurgence (*p* = 0.0032) observed in 2022, with male and female rates both reaching 386.4 and 386.3 surgeries per 100,000 inhabitants, respectively. The incidence is expected to slowly drop on a national level during the coming years. By 2035 the incidence of knee surgeries is expected to be 172, CI: 151–202, for men and 249, CI: 224–278, for women (Figure 1). The ratio between female and male surgical rates remained constant and did not change significantly from 2010 to 2022 (*p* = 0.53).

**Figure 2:**
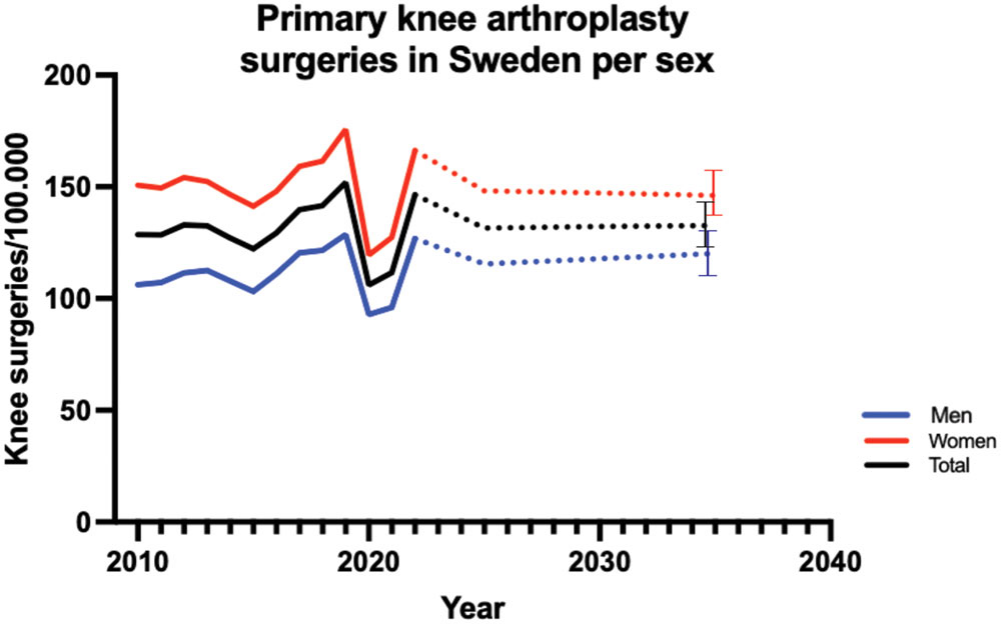
Primary knee arthroplasty surgeries in Sweden. The dotted line indicates future trend. Bars indicate 95% CI. CI, confidence interval.

**Figure 3:**
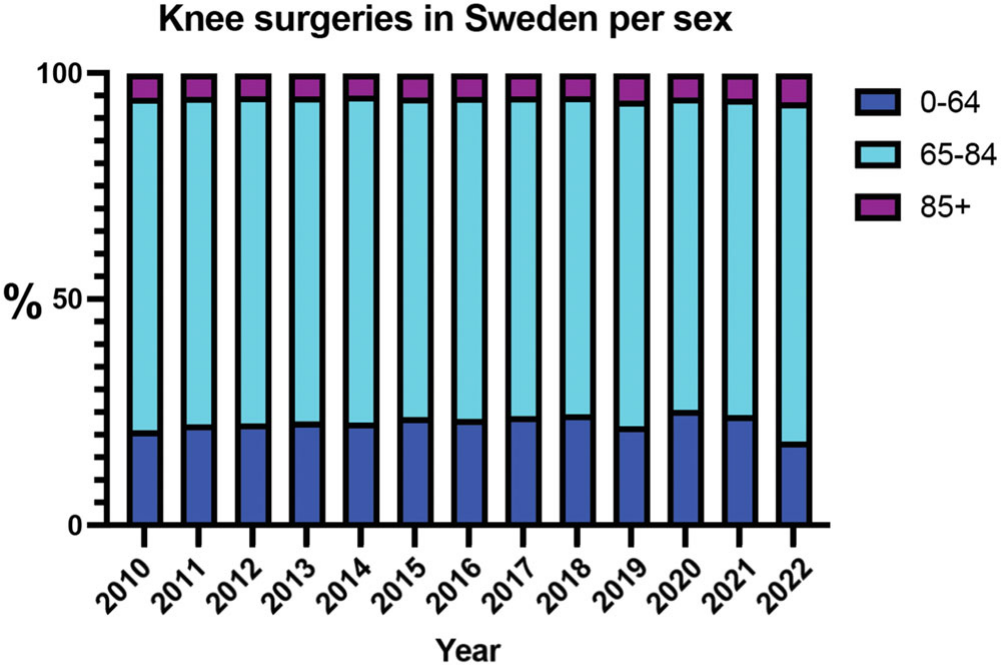
Age groups undergoing primary knee arthroplasty surgery.

The general surgical category of primary knee arthroplasty surgeries displayed dynamic trends. In 2010, the male rate was 106.2 surgeries per 100,000 inhabitants, while the female rate was 150.7. These rates exhibited fluctuations but showed an overall upward trajectory, peaking in 2019. By 2022, the male rate reached 126.8, and the female rate was 166.2 surgeries per 100,000 inhabitants. The incidence of primary knee arthroplasty is expected to remain constant during the coming years. By 2035, the incidence of primary knee arthroplasty is expected to be 120, CI: 109–131, for men and 146, CI: 136–156, for women (Figure 2). Age groups undergoing knee arthroplasty surgery remained relatively constant during the study period, with no significant shift towards more elderly groups during 2020–2022 (*p* = 0.31) (Figure 3).

This study examined unicondylar and total knee arthroplasty using cement and cementless techniques. Incidence of primary unicondylar knee arthroplasty without cement experienced a gradual increase for both genders, with the rate in 2022 at 11.8 surgeries per 100,000 inhabitants. There is an expected increase in the incidence of unicondylar arthroplasty without cement during the coming years. Rates of primary unicondylar arthroplasty with cement remained relatively stable, with a rate of 5.3 surgeries per 100,000 inhabitants in 2022 and no expected fluctuations in incidence during the coming years. Women and men experienced similar incidences regardless of surgical technique (Figure S1).

**Figure 4:**
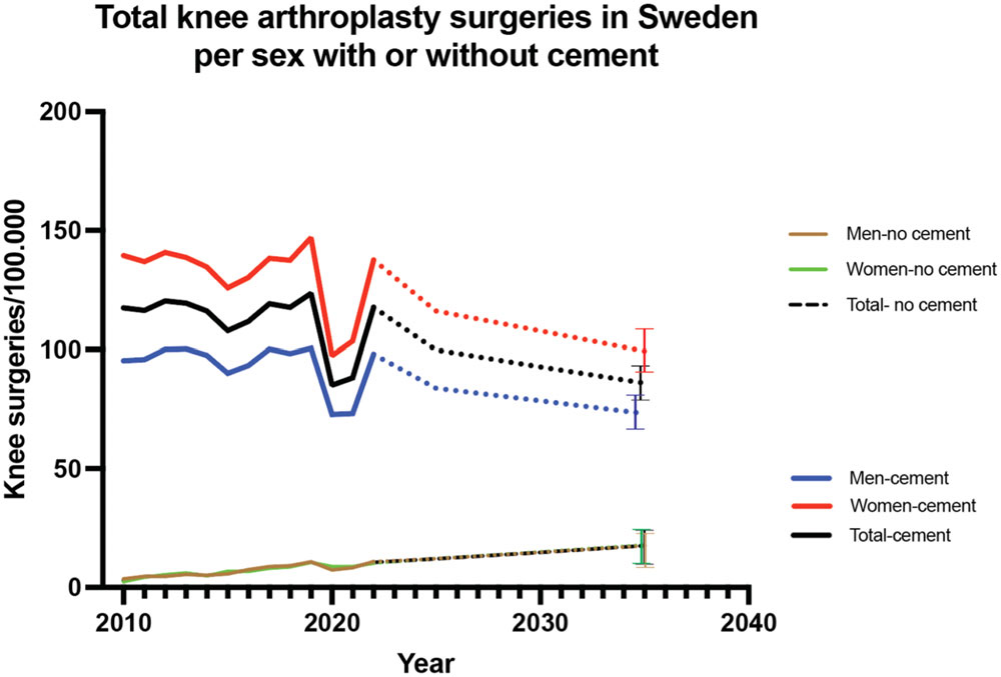
Total knee arthroplasty surgeries in Sweden per sex, with or without cement. Dotted line indicates future trend. Bars indicate 95% CI. CI, confidence interval.

Primary total knee arthroplasty had a rate of 117.7 surgeries per 100,000 inhabitants in 2022. There was a significant drop during the COVID‐19 pandemic years of 2020–2022 (*p* = 0.0021). Trend projection showed a decreased incidence of total knee arthroplasty incidence with cement during the coming years. The trend for the incidence of total knee arthroplasty with cement will increase slightly in the coming years. By 2035, the overall incidence of total knee arthroplasty is expected to be 73, CI: 68–82, for men and 99, CI: 90–108, for women undergoing total knee arthroplasty with cement and 17, CI: 12–22, for men and 18, CI: 13–23, for women who have undergone total knee arthroplasty without cement (Figure 4).

Primary total knee arthroplasty with hybrid technology remained fairly rare as a surgical technique, with 0.6 surgeries per 100,000 inhabitants in 2022. Trend projection indicated a stable or slightly decreasing incidence, primarily driven by a decrease among women. By 2035, the incidence is expected to be 0.6, CI: 0.4–0.8, for men and 0.3, CI 0.1–0.5, for women (Figure S2).

**Figure 5:**
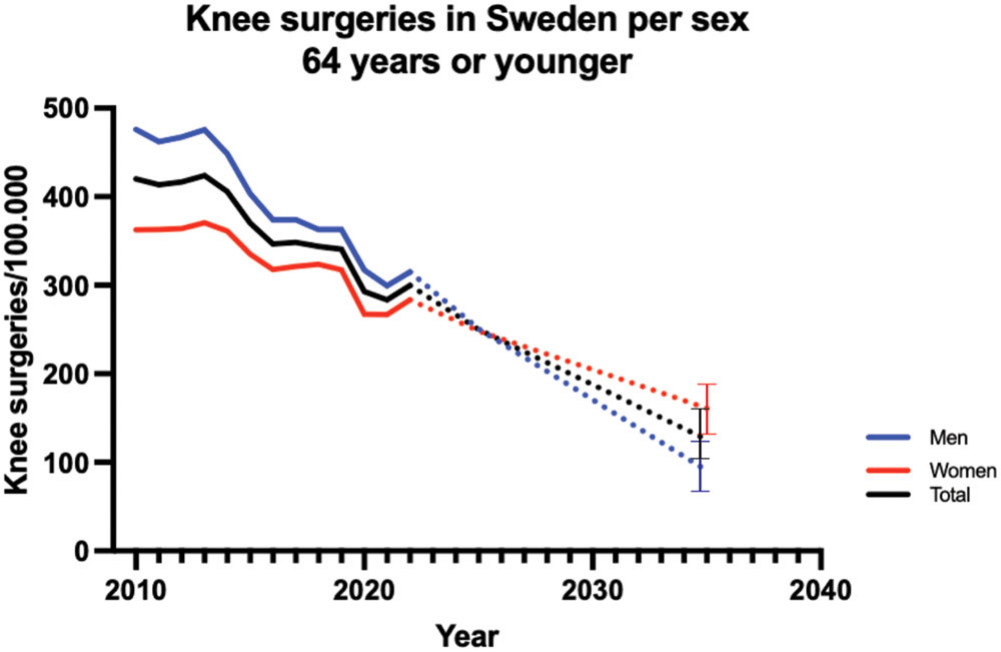
Knee surgeries in people aged 64 years or younger. Bars indicate 95% CI. CI, confidence interval.

In 2010, the surgical rate for individuals aged 64 or younger was 475.9 procedures per 100,000 inhabitants for males and 362.4 for females. These rates reached 299.4 for males and 283.5 for females in 2022. When considering both men and women aged 64 and younger, the combined surgical rates showed a decline. In 2010, the surgical rate for knee and lower leg procedures for this age group was 420.2 per 100,000 inhabitants. This rate decreased to 299.7 in 2022. By 2035, the incidence is expected to be 91, CI: 70–112, for men and 161, CI: 130–192, for women (Figure 5).

**Figure 6:**
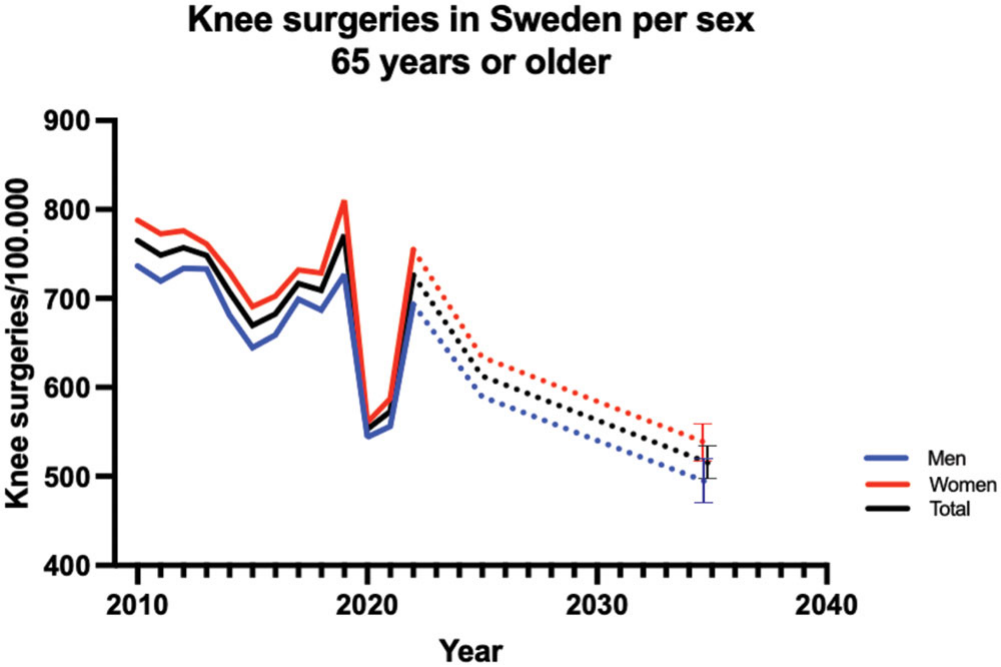
Knee surgery per sex and age group in Sweden 2010–2022. Bars indicate 95% CI. CI, confidence interval.

For men and women aged 65 and older, the combined surgical rates exhibit a pattern of stability followed by a decline and subsequent recovery. In 2010, the surgical rate for knee procedures for this age group was 764.9 per 100,000 inhabitants. This rate remained relatively stable until 2019 when it had a significant drop to 553.0 (*p* = 0.0094), after which it increased to 726.0 in 2022. In 2010, the surgical rate for elderly males was 736.5 per 100,000 inhabitants. This rate remained relatively stable until 2019, when it saw a significant decline (*p* = 0.0044), reaching 544.0 in 2020. There was a rebound in 2022 with a surgical rate of 693.0. For elderly females, a similar pattern is observed. The surgical rate was 787.8 in 2010, showing a gradual decline until 2020 (560.8). However, like their male counterparts, there was an increase in 2022, with a surgical rate of 755.0. By 2035, the incidence is expected to be 491, CI: 465–491, for men and 535, CI: 515–555, for women (Figure 6).

## DISCUSSION

The most important finding of this study is that the COVID‐19 pandemic significantly impacted knee surgery rates in Sweden, causing a notable decline in 2020, followed by a resurgence in 2022. Furthermore, while men had higher surgery rates than women, they experienced a larger decline in the incidence of knee surgeries compared to women. Our hypothesis that knee surgery incidence was increasing during the last years was not accepted, although knee arthoplasty incidence did increase during the study period.

This underscores the pandemic's disruption of elective procedures and highlights the healthcare system's resilience and adaptability in the face of such disruptions. These findings are critical for future healthcare planning to ensure continuity and accessibility of surgical care.

The fluctuations in surgical rates observed during the study period reflect the changeable nature of healthcare delivery, which has changed notably during the last decade. Our findings are novel as a national study on knee surgery and sub‐procedures using open‐source data has not before been conducted in Sweden, nor have the effects of the COVID‐19 pandemic been analysed in regard to knee surgery and affected patient populations. A regional study in Southern Sweden found significant disturbances in elective knee surgery incidence [6]. As expected, the most notable change was the decline in surgical rates in 2020, attributed to the disruptions caused by the COVID‐19 pandemic. This finding underscores the vulnerability of elective surgeries to external factors. A similar trend has been noted in other countries [3, 5, 31]. In particular, in regard to elective surgeries [4, 7]. This decline, followed by a resurgence in 2022, highlights the need for healthcare systems to adapt and ensure the provision of timely surgical interventions even in challenging circumstances.

Sex‐specific variations in surgical rates have implications for healthcare equity. While males consistently exhibited higher surgical rates than females, our study found that both genders experienced fluctuations and shared in the resurgence observed in 2022. The reasons behind these disparities warrant further investigation, as they may be influenced by factors such as differences in the prevalence of knee conditions, healthcare‐seeking behaviours, or physician referral patterns. This is important due to recently described differences in patient outcomes following elective knee surgeries and the implication of surgeon bias. Physicians might be more likely to perceive worse symptomatology in women compared to men prior to elective surgery which might influence surgery decisions [18]. Although pain levels, functionality, and symptoms do not appear to differ between men and women post‐surgery [2]. The use of gender‐specific pain instruments and indications might be useful tools for orthopaedic surgeons in the evaluation of patients for knee arthroplasty [15].

The upward trend in primary knee arthroplasty surgeries is indicative of the growing importance of these procedures in addressing knee‐related conditions. The year 2019 marked a peak in surgical rates, suggesting an increasing demand for knee joint replacement surgeries. This could be attributed to the ageing population, greater awareness of knee conditions, and advancements in surgical techniques. A conclusion that has been described by other studies [11, 28]. Our future projection analysis for primary knee arthroplasty supports this conclusion and indicates stable incidence rates, with 120 surgeries per 100,000 men and 146 surgeries per 100,000 women by 2035. This stability suggests persistent demand due to the prevalence of degenerative joint conditions among the elderly [9, 30].

Although knee arthroplasty remains popular, orthopaedic surgeons and healthcare providers should be aware of changing trends in knee surgeries. Recent data indicate that patients are becoming slightly older, compared to the previously uniform age distribution. Additionally, there is a shift in procedural techniques: hybrid techniques are becoming less common among women, while cement‐free arthroplasty procedures are increasing in incidence among both men and women. Our analysis of future incidence of subprocedures shows that the incidence of primary unicondylar knee arthroplasty is expected to increase while primary total knee arthroplasty is expected to decrease by 2035. We also noted an anticipated increase in total knee arthroplasty without cement, which suggests a potential shift towards cementless techniques. As mentioned, these trends likely reflect advancements in surgical technologies and evolving preferences among both patients and surgeons. For example, unicondylar knee arthroplasty is associated with shorter hospital stays and fewer complications, potentially prompting their increased use [10, 13]. Cementless knee arthroplasty has been suggested to have better long‐term outcomes in some studies [20, 23]. A recent meta‐analysis also found that long‐term recovery was better in patients who had undergone cementless knee arthroplasty [16]. However, further research is needed to fully understand the factors influencing these choices and their implications for patient outcomes.

Trends in primary knee arthroplasty also differ between countries. Studies from the United States and Italy show a more aggressive growth in the trend of primary knee arthroplasty with estimates ranging from 5% to 7% annually [14, 27]. A study by Erivan et al. reported an increase of 32% in primary knee arthroplasty in France between 2012 and 2018 [8]. A study in South Korea found an increase in primary knee arthroplasty of roughly 4% annually from 2010 to 2018 [12]. A retrospective German study found that the increase in primary knee arthroplasty was heavily dependent on age and procedure type while showing an increase of barely 12% from 2008 to 2017 [34]. In comparison, our study found the increase in primary knee arthroplasty to be approximately 20% during the study period with an annual increase of roughly 2%. These variances between countries are likely indicative of changes under underlying patient factors such as obesity and age. Furthermore, the development of implants and instruments and better surgical techniques are significant drivers of surgery trends which might vary between countries [1].

The data analysed is subject to the accuracy and completeness of healthcare records, and the study does not delve into individual patient‐level factors or outcomes, making our analysis crude. Additionally, while the data provide insights into surgical rates, it does not offer explicit explanations for the observed trends, which may require further qualitative or contextual analysis. Finally, underestimation of data variance might be an issue based on our dispersion of variables and should be considered when interpreting our results. Nevertheless, our findings emphasize the need for robust healthcare planning and adaptation. Monitoring surgical trends and understanding their drivers are critical for healthcare policymakers and providers. Additionally, this is to our knowledge the first national‐wide trend analysis study on knee surgery trends, which considers sub‐procedures, age and sex, using open‐source data in the Swedish population.

## CONCLUSION

This nationwide analysis of knee surgeries in Sweden from 2010 to 2022 reveals significant gender disparities and the impact of the COVID‐19 pandemic on surgical trends. Men and women younger than 65 years of age were particularly affected by decreases in surgery incidence, and stakeholders should monitor future surgery trends in order to manage increasing demand in elective procedures following the pandemic.

## AUTHOR CONTRIBUTIONS

**Michael Axenhus** and **Martin Magnéli:** Conceptualization; data curation; formal analysis; investigation; methodology; software; validation. **Michael Axenhus:** Project administration; funding acquisition; supervision; visualization; writing—original draft. **Martin Magnéli:** Resources; writing—review and editing.

## CONFLICT OF INTEREST STATEMENT

The authors declare no conflict of interest.

## ETHICS STATEMENT

The data used in this study are obtained from the website of the SNBHW and are publicly available for anyone to download and use.

## Supporting information

Supporting information.

Supporting information.

Supporting information.

## Data Availability

The raw data sets are available in the FigShare depository (https://figshare.com/s/df5678ba3488b6fa67ea).
