# Supplementary material for: The COVID‐19 pandemic caused gender‐specific declines in knee surgery rates in Sweden from 2020 to 2021
Source: J Exp Orthop. 2024 Oct 3;11(4):e70030. doi: 10.1002/jeo2.70030 (PMC11447367; doi:10.1002/jeo2.70030)
Supplement: Supplementary file 2 — Supporting information. [file JEO2-11-e70030-s002.pdf]

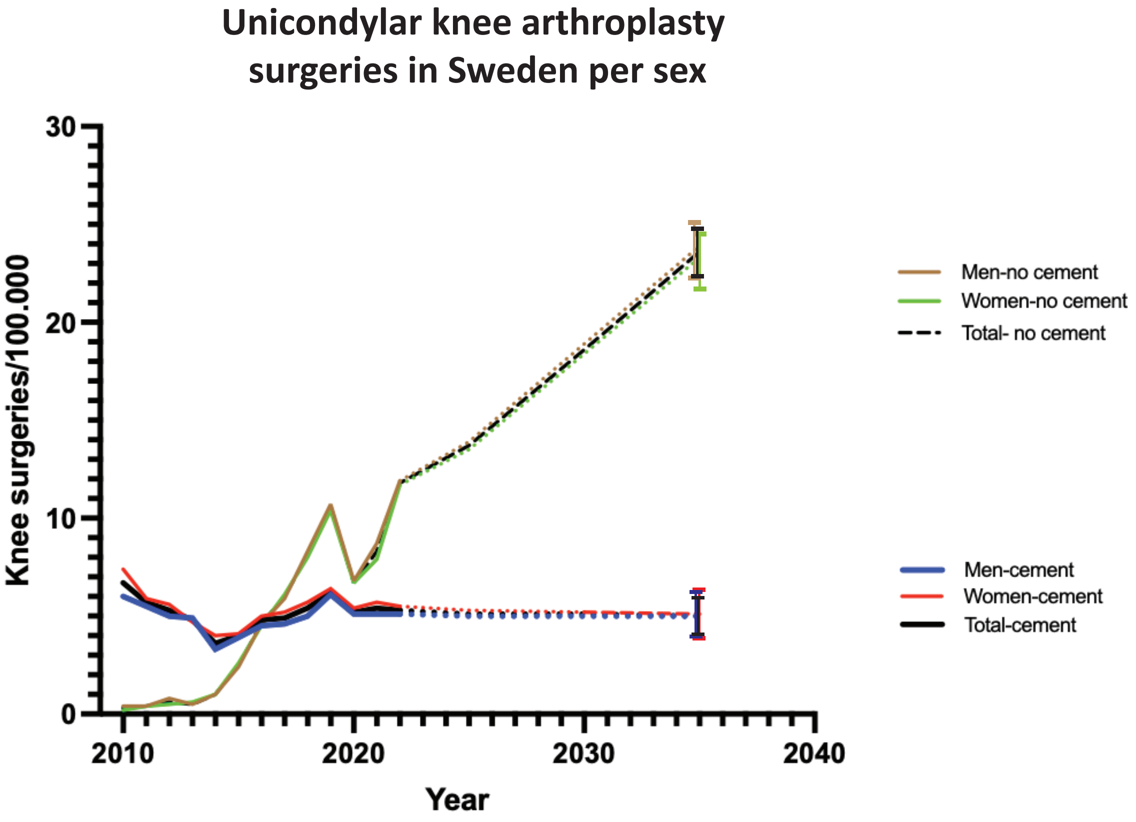

Supplemental figure 1: Unicondylar arthroplasty surgeries in Sweden per sex, with or without cement. Dotted line indicates future trend. Bars indicate 95% CI.
